# Supplementary material for: Perturbation of TM6SF2 Expression Alters Lipid Metabolism in a Human Liver Cell Line
Source: Int J Mol Sci. 2021 Sep 9;22(18):9758. doi: 10.3390/ijms22189758 (PMC8471112; doi:10.3390/ijms22189758)
Supplement: Supplementary file 1 [file ijms-22-09758-s001.zip › ijms-1363644-supplementary.pdf]

Supplementary material

Supplementary Figure S1

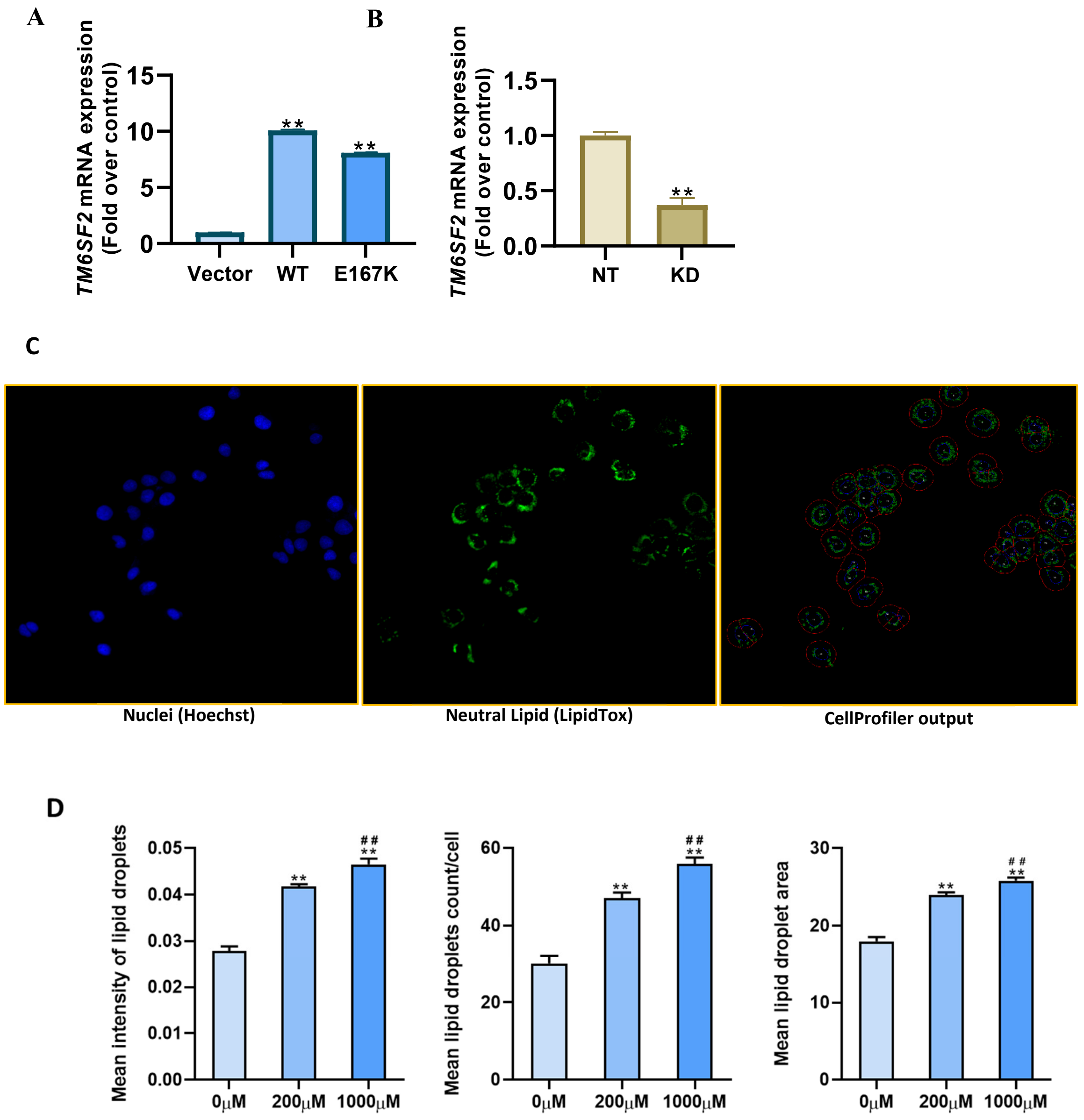

Supplementary Figure S1: Validation of *TM6SF2* gene knockdown and *TM6SF2* overexpression in Huh-7 cells. Quantitation of *TM6SF2* mRNA expression using RT-qPCR in (**A**) overexpression and (**B**) knockdown Huh-7 cell lines. (**C**) Microscopic image of cell nuclei stained with Hoechst (Blue; left panel) and neutral lipids by LipidTox Green (green; center panel). Right panel shows the areas for nuclei (blue circle), lipid droplets (green circles), and cell perimeter (red circle) identified by the CellProfiler pipeline. (**D**) Dose-dependent effects of oleic acid treatment on different lipid droplet variables in Vector control cells. For bar graphs, data is presented as mean+ S.E.M. from 3 independent experiments, ** significantly different than control, $P < 0.01$. $^{\#\#}$ significantly different than 200 μM oleic acid $P < 0.01$. Wild-type *TM6SF2* (WT), E167K mutant variant *TM6SF2* (E167K), pCMV empty vector control (Vector), non-targeted shRNA control (NT), and *TM6SF2* knockdown (KD).

Supplementary Figure S2: Unsupervised Hierarchical clustering analysis for 350 lipid species.

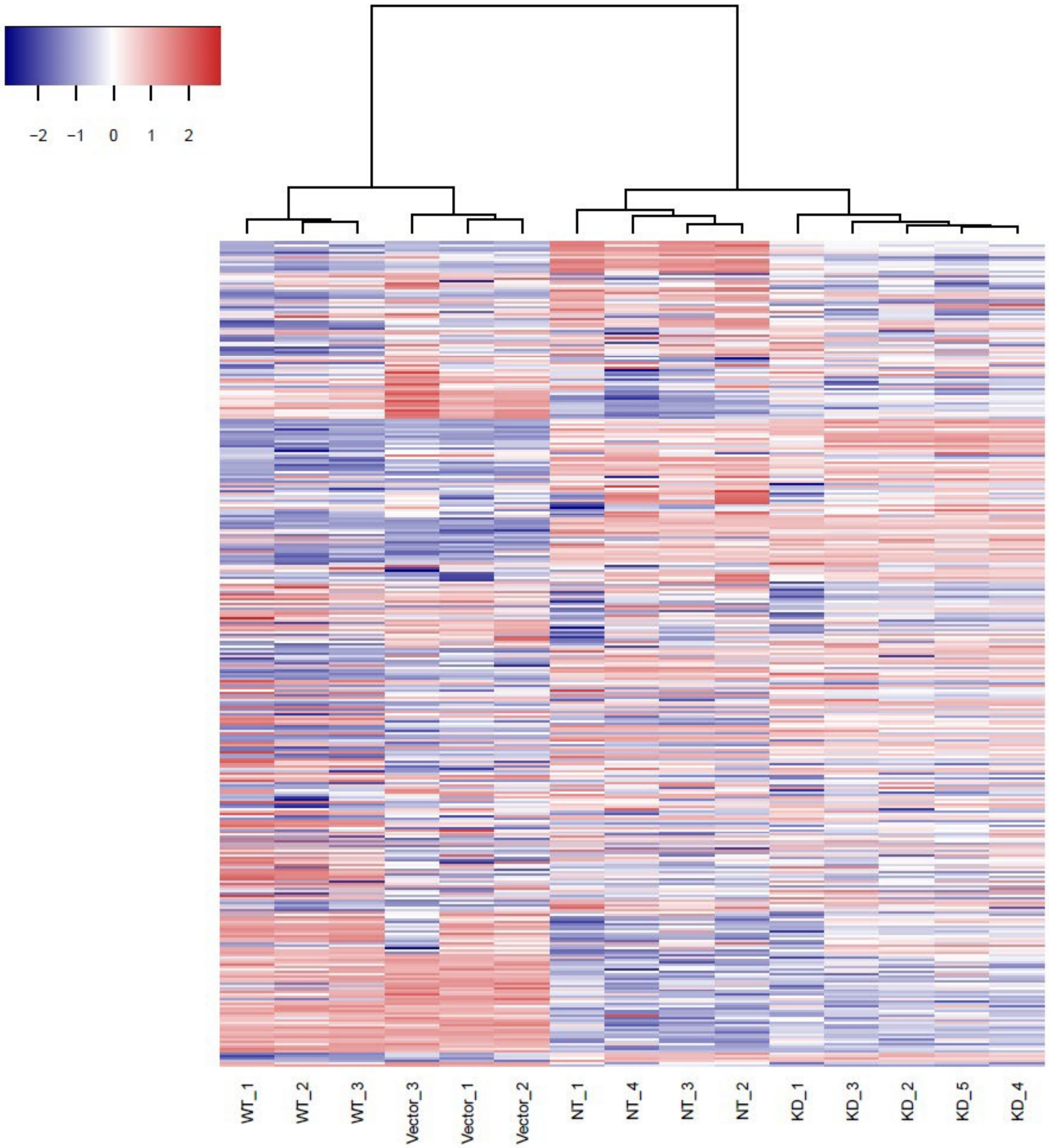

Supplementary Figure S2: Red color represents the abundance of each lipid species (red: higher, blue: lower). WT (wild-type *TM6SF2* overexpression, Vector (empty vector control), NT (Non-targeted shRNA control), and KD (*TM6SF2* knockdown). Numbers at the ends refers to each replicate.

Supplementary Figure S3: Heatmap of side chain length and degree of unsaturation for lipid species identified in the lipidomic analysis.

A

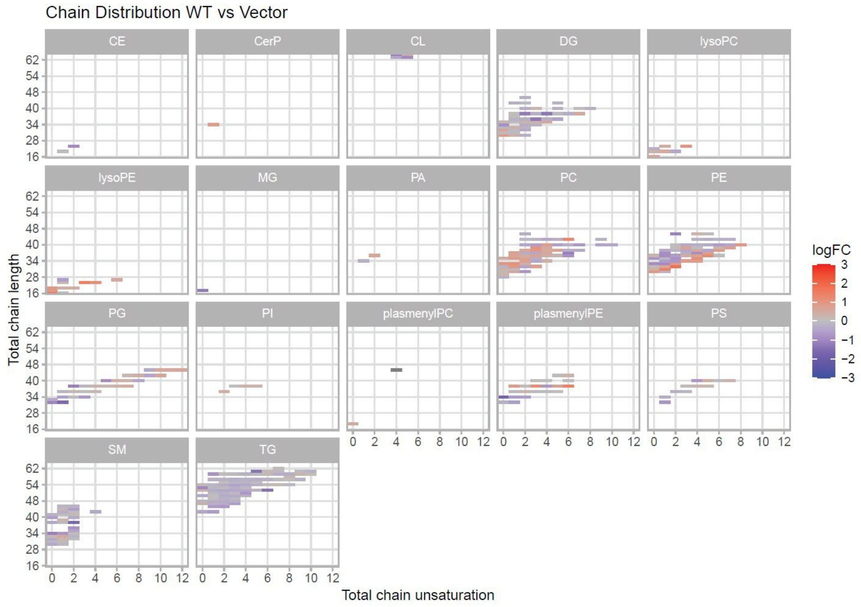

B

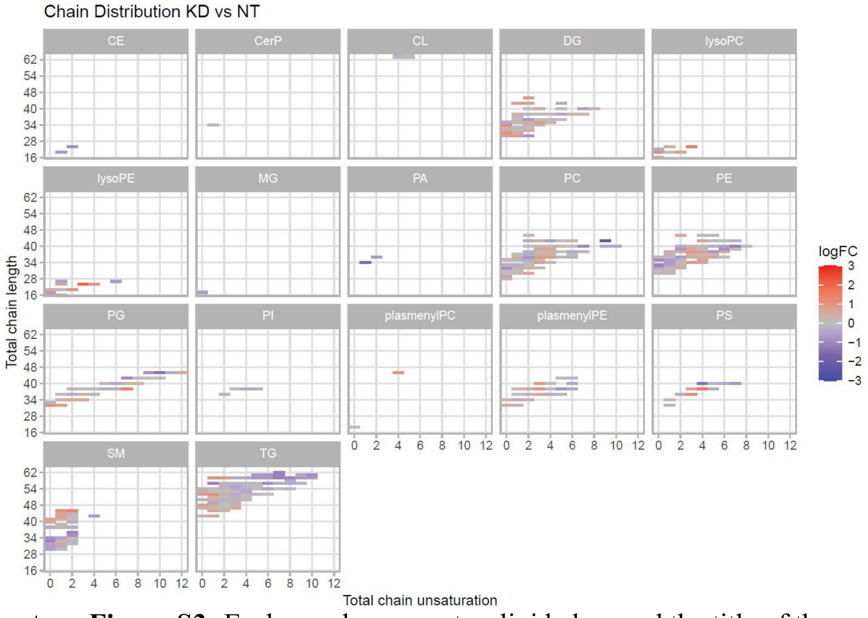

Supplementary Figure S3: Each panel represents a lipid class and the title of the panel is the abbreviated name of the lipid class. Each box represents an individual lipid species where the colors indicate the relative ratio of the lipid (red: increased; white: no difference; blue: decreased) in (A) wild-type *TM6SF2* overexpression (n=3) compared to vector control (n=3) and (B) *TM6SF2* knockdown (n=5) versus non targeting control (n=4). CE (cholesterol esters), CerP (ceramide 1-phosphate), CL (Cardiolipin), DG (diacylglycerols), lysoPC (Lysophosphatidylcholines), lysoPE (lysophosphatidylethanolamine), MG (monoacylglycerols), PA (Phosphatidic acid), PC (phosphatidylcholine), PE (Phosphatidylethanolamine), PG (Phosphatidylglycerol), PI (phosphatidylinositol), plasmenylPC (Plasmenylphosphatidylcholines), plasmenylPE (Phosphatidylethanolamines), PS (Phosphatidylserines), SM (Sphingomyelines), TG (Triacylglycerols).WT (wild-type *TM6SF2* overexpression, Vector (empty vector control), NT (Non-targeted shRNA control), and KD (*TM6SF2* knockdown).

Supplementary Table S1: List of differentially abundant lipid species identified through untargeted lipidomic analysis.

Comparison	Molecule	Class	P.Val	Fold Change	LogFC
**KD - NT**	DG 31:0	DG	0.00985	2.09	1.06
**KD - NT**	DG 34:0	DG	0.01193	2.35	1.23
**KD - NT**	lysoPC 18:0	lysoPC	0.00007	2.33	1.22
**KD - NT**	lysoPC 20:3	lysoPC	0.00007	3.03	1.60
**KD - NT**	lysoPE 16:0	lysoPE	0.00433	2.01	1.00
**KD - NT**	lysoPE 18:2	lysoPE	0.00345	2.01	1.01
**KD - NT**	lysoPE 20:3	lysoPE	0.00007	3.99	2.00
**KD - NT**	PA 34:1	PA	0.01258	-2.92	-1.55
**KD - NT**	PC 42:9	PC	0.00262	-4.63	-2.21
**KD - NT**	PC 40:7	PC	0.00013	-2.05	-1.03
**KD - NT**	PE 40:7	PE	0.00011	-2.22	-1.15
**KD - NT**	PE 33:0	PE	0.00099	-2.07	-1.05
**KD - NT**	PG 44:10	PG	0.00024	-2.59	-1.38
**KD - NT**	PG 32:0	PG	0.01088	2.49	1.31
**KD - NT**	PG 38:7	PG	0.00007	2.70	1.43
**KD - NT**	plasmenylPE 38:3	plasmenylPE	0.00007	2.04	1.03
**KD - NT**	PS 40:4	PS	0.00066	-2.69	-1.43
**KD - NT**	PS 36:3	PS	0.00619	2.28	1.19
**KD - NT**	PS 38:4	PS	0.00046	3.12	1.64
**KD - NT**	SM 33:0	SM	0.00007	-2.10	-1.07
**KD - NT**	SM 36:2	SM	0.00013	-2.04	-1.03
**KD - NT**	SM 44:1	SM	0.00324	2.11	1.08
**KD - NT**	SM 44:2	SM	0.00020	2.60	1.38
**KD - NT**	TG 56:1	TG	0.00319	-2.23	-1.16
**KD - NT**	TG 62:7	TG	0.00007	-2.18	-1.12
**KD - NT**	TG 60:7	TG	0.00007	-2.14	-1.10
**KD - NT**	TG 58:7	TG	0.00008	-2.13	-1.09
**KD - NT**	TG 58:8	TG	0.00007	-2.08	-1.06
**KD - NT**	TG 58:1	TG	0.00016	2.13	1.09
**KD - NT**	TG 52:1	TG	0.01003	2.30	1.20
**WT - Vector**	CL 72:4	CL	0.02068	-2.31	-1.21
**WT - Vector**	DG 38:4	DG	0.00272	-2.46	-1.30
**WT - Vector**	DG 36:3	DG	0.00272	-2.44	-1.28
**WT - Vector**	DG 38:2	DG	0.00390	-2.35	-1.23
**WT - Vector**	lysoPE 20:4	lysoPE	0.00666	2.21	1.14
**WT - Vector**	lysoPE 16:0	lysoPE	0.03878	2.74	1.46
**WT - Vector**	lysoPE 20:3	lysoPE	0.01081	2.87	1.52
**WT - Vector**	MG 17:0	MG	0.00390	-2.40	-1.26
**WT - Vector**	PC 37:6	PC	0.02634	-2.25	-1.17
**WT - Vector**	PC 39:3	PC	0.00721	2.04	1.03
**WT - Vector**	PC 33:1	PC	0.00272	2.14	1.10
**WT - Vector**	PC 42:6	PC	0.00882	2.45	1.29
**WT - Vector**	PE 30:0	PE	0.00305	2.00	1.00

Comparison	Molecule	Class	P.Val	Fold Change	LogFC
**WT - Vector**	PE 35:4	PE	0.00354	2.21	1.15
**WT - Vector**	PE 31:1	PE	0.00199	2.82	1.50
**WT - Vector**	PE 32:2	PE	0.00199	3.40	1.76
**WT - Vector**	PG 32:1	PG	0.00305	-2.96	-1.57
**WT - Vector**	PG 38:2	PG	0.00272	-2.25	-1.17
**WT - Vector**	PG 32:0	PG	0.04293	-2.08	-1.06
**WT - Vector**	plasmenylPC 44:4	plasmenylPC	0.03364	10.85	3.44
**WT - Vector**	plasmenylPE 34:0	plasmenylPE	0.00305	-3.46	-1.79
**WT - Vector**	plasmenylPE 38:3	plasmenylPE	0.00438	2.24	1.16
**WT - Vector**	plasmenylPE 38:6	plasmenylPE	0.01815	2.72	1.44
**WT - Vector**	SM 38:2	SM	0.03085	-3.07	-1.62
**WT - Vector**	TG 52:6	TG	0.00272	-2.71	-1.44
**WT - Vector**	TG 60:5	TG	0.00418	-2.65	-1.41

Supplementary Table S 1: Only the lipid species that were differentially abundant (adjusted p value < 0.05 and log fold change > 0.5 or < -0.5) in cells with either wild-type *TM6SF2* overexpression or knockdown compared to their controls (vector or non-targeted controls respectively) are shown. WT (wild-type *TM6SF2* overexpression), Vector (empty vector control), NT (Non-targeted shRNA control), and KD (*TM6SF2* knockdown). Lipid species that were changed across two comparisons belonged to the following classes: DG (diacylglycerols), lysoPC (Lysophosphatidylcholines), lysoPE (lysophosphatidylethanolamine), MG (monoacylglycerols), PA (Phosphatidic acid), PC (phosphatidylcholine), PE (Phosphatidylethanolamine), PG (Phosphatidylglycerol), plasmenylPC (Plasmenylphosphatidylcholines), plasmenylPE (Phosphatidylethanolamines), PS (Phosphatidylserines), SM (Sphingomyelines), TG (Triacylglycerols).

Supplementary Table S2: Alterations in *TM6SF2* expression results in differentially expressed lipid metabolizing genes.

Comparison	Symbol	Protein names	P. Val	LogFC	Fold Change
**WT - Vector**	ALDH3B1	Aldehyde dehydrogenase family 3 member B1	1.00E-06	1.37	2.58
**WT - Vector**	APOD	Apolipoprotein D	1.00E-06	2.02	4.05
**WT - Vector**	RGL1	Ral guanine nucleotide dissociation stimulator-like 1	6.39E-06	0.58	1.49
**WT - Vector**	DAB2IP	Disabled homolog 2-interacting protein	6.54E-06	0.84	1.79
**WT - Vector**	AGTR1	Type-1 angiotensin II receptor	2.45E-05	-1.21	0.43
**WT - Vector**	LIPH	Lipase member H	7.58E-05	1.10	2.14
**WT - Vector**	AKR1C1	Aldo-keto reductase family 1 member C1	9.92E-05	-1.36	0.39
**WT - Vector**	VAV3	Guanine nucleotide exchange factor	0.00071	0.70	1.63
**WT - Vector**	AGMO	Alkylglycerol monooxygenase	0.00336	-0.65	0.64
**WT - Vector**	AKR1C4	Aldo-keto reductase family 1 member C4	0.00373	-0.98	0.51
**WT - Vector**	FDFT1	Squalene synthase	0.00382	-0.50	0.70
**WT - Vector**	CAPN2	Calpain-2 catalytic subunit	0.00478	1.17	2.25
**WT - Vector**	APOA2	Apolipoprotein A-II	0.00517	-0.58	0.67
**WT - Vector**	HSD17B2	17-beta-hydroxysteroid dehydrogenase type 2	0.00778	0.53	1.45
**WT - Vector**	AKR1C2	Aldo-keto reductase family 1 member C2	0.02397	-1.13	0.46
**WT - Vector**	TM6SF2	Transmembrane 6 superfamily member 2	1.00E-06	1.75	3.37
**KD - NT**	ACAA1	3-ketoacyl-CoA thiolase	1.00E-06	0.81	1.75
**KD - NT**	AKR1B1	Aldo-keto reductase family 1 member B1	1.00E-06	2.73	6.64
**KD - NT**	ANXA1	Annexin A1	1.00E-06	3.79	13.87
**KD - NT**	BMP2	Bone morphogenetic protein 2	1.00E-06	0.94	1.91
**KD - NT**	CYP2C19	Cytochrome P450 2C19	1.00E-06	-2.13	0.23
**KD - NT**	EHHADH	Peroxisomal bifunctional enzyme	1.00E-06	-0.65	0.64
**KD - NT**	CCN1	CCN family member 1	1.00E-06	1.73	3.32
**KD - NT**	MVD	Diphosphomevalonate decarboxylase	1.00E-06	0.66	1.58
**KD - NT**	PDGFRA	Platelet-derived growth factor receptor alpha	1.00E-06	-1.20	0.43
**KD - NT**	PPARG	Peroxisome proliferator-activated receptor gamma	1.00E-06	0.69	1.62
**KD - NT**	PTGIS	Prostacyclin synthase	1.00E-06	-1.03	0.49
**KD - NT**	THRA	Thyroid hormone receptor alpha	1.00E-06	-1.15	0.45
**KD - NT**	UBE2I	SUMO-conjugating enzyme UBC9	1.00E-06	0.83	1.78
**KD - NT**	VDR	Vitamin D3 receptor	1.00E-06	1.66	3.16
**KD - NT**	PIK3R3	Phosphatidylinositol 3-kinase regulatory subunit gamma	1.00E-06	0.89	1.85
**KD - NT**	DGAT1	Diacylglycerol O-acyltransferase 1	1.00E-06	-0.88	0.55
**KD - NT**	ABCC3	ATP-binding cassette sub-family C member 3	1.00E-06	1.15	2.22
**KD - NT**	LPCAT3	Lysophospholipid acyltransferase 5	1.00E-06	0.54	1.45
**KD - NT**	VAV3	Guanine nucleotide exchange factor	1.00E-06	2.22	4.65
**KD - NT**	RGL1	Ral guanine nucleotide dissociation stimulator-like 1	1.00E-06	0.97	1.96
**KD - NT**	ANKRD1	Ankyrin repeat domain-containing protein 1	1.00E-06	3.56	11.80
**KD - NT**	TNFRSF21	Tumor necrosis factor receptor superfamily member 21	1.00E-06	0.96	1.95
**KD - NT**	ANGPTL3	Angiopoietin-related protein 3	1.00E-06	1.05	2.08
**KD - NT**	ARSJ	Arylsulfatase J	1.00E-06	1.20	2.30
**KD - NT**	ELOVL7	Elongation of very long chain fatty acids protein 7	1.00E-06	1.26	2.39
**KD - NT**	DGAT2	Diacylglycerol O-acyltransferase 2	1.00E-06	0.67	1.59
**KD - NT**	SLC44A3	Choline transporter-like protein 3	1.00E-06	1.22	2.33
**KD - NT**	TTC39B	Tetratricopeptide repeat protein 39B	1.00E-06	-1.32	0.40

Comparison	Symbol	Protein names	P. Val	LogFC	Fold Change
**KD - NT**	AKR1C4	Aldo-keto reductase family 1 member C4	1.45E-06	1.48	2.79
**KD - NT**	SLCO1B3	Solute carrier organic anion transporter family member 1B3	1.69E-06	-1.35	0.39
**KD - NT**	EPHX2	Bifunctional epoxide hydrolase 2	1.91E-06	-0.60	0.66
**KD - NT**	SGMS1	Phosphatidylcholine:ceramide cholinephosphotransferase 1	3.33E-06	0.82	1.77
**KD - NT**	STUB1	E3 ubiquitin-protein ligase	6.40E-06	0.71	1.64
**KD - NT**	ABCC1	Multidrug resistance-associated protein 1	7.89E-06	0.87	1.83
**KD - NT**	LIPH	Lipase member H	7.89E-06	1.33	2.51
**KD - NT**	ACBD4	Acyl-CoA-binding domain-containing protein 4	1.83E-05	-0.70	0.61
**KD - NT**	B4GALNT1	Beta-1,4 N-acetylgalactosaminyltransferase 1	2.03E-05	0.50	1.42
**KD - NT**	HMGCS2	Hydroxymethylglutaryl-CoA synthase	2.57E-05	0.98	1.98
**KD - NT**	LRP2	Low-density lipoprotein receptor-related protein 2	2.84E-05	-0.75	0.60
**KD - NT**	GGT1	Glutathione hydrolase 1 proenzyme	2.85E-05	-0.95	0.52
**KD - NT**	PLA2G4A	Cytosolic phospholipase A2	2.92E-05	-1.05	0.48
**KD - NT**	UGT1A1	UDP-glucuronosyltransferase 1A1	4.09E-05	1.64	3.13
**KD - NT**	HPGD	15-hydroxyprostaglandin dehydrogenase	6.18E-05	1.18	2.26
**KD - NT**	STARD10	START domain-containing protein 10	0.000117	-0.54	0.69
**KD - NT**	SPTSSA	Serine palmitoyltransferase small subunit A	0.000153	-0.71	0.61
**KD - NT**	PON3	Serum paraoxonase/lactonase 3	0.000199	-0.85	0.55
**KD - NT**	B3GALNT1	UDP-GalNAc:beta-1,3-N-acetylgalactosaminyltransferase 1	0.000271	0.61	1.52
**KD - NT**	PPARGC1B	Peroxisome proliferator-activated receptor gamma coactivator 1-beta	0.000291	-0.61	0.66
**KD - NT**	LPCAT4	Lysophospholipid acyltransferase LPCAT4	0.000433	-0.77	0.59
**KD - NT**	GDPD1	Lysophospholipase D GDPD1	0.000553	-0.55	0.68
**KD - NT**	SLC27A2	Very long-chain acyl-CoA synthetase	0.000665	-0.85	0.55
**KD - NT**	OSBPL1A	Oxysterol-binding protein-related protein 1	0.000748	-0.74	0.60
**KD - NT**	CYP2R1	Vitamin D 25-hydroxylase	0.000748	-0.58	0.67
**KD - NT**	DAB2IP	Disabled homolog 2-interacting protein	0.000832	-0.65	0.64
**KD - NT**	CAPN2	Calpain-2 catalytic subunit	0.000931	1.31	2.48
**KD - NT**	ACP6	Lysophosphatidic acid phosphatase type 6	0.000962	-0.53	0.69
**KD - NT**	SEC14L2	SEC14-like protein 2	0.001312	-0.84	0.56
**KD - NT**	APOA5	Apolipoprotein A-V	0.001321	-1.12	0.46
**KD - NT**	TGFB1	Transforming growth factor beta-1 proprotein	0.001356	-0.90	0.54
**KD - NT**	LSR	Lipolysis-stimulated lipoprotein receptor	0.001498	0.51	1.43
**KD - NT**	PLIN5	Perilipin-5	0.001606	-0.90	0.54
**KD - NT**	SLCO1A2	Solute carrier organic anion transporter family member 1A2	0.001779	-0.63	0.65
**KD - NT**	G0S2	G0/G1 switch protein 2	0.002284	-0.91	0.53
**KD - NT**	SPHK1	Sphingosine kinase 1	0.002309	-0.53	0.69
**KD - NT**	SIRT3	NAD-dependent protein deacetylase sirtuin-3	0.002604	-0.51	0.70
**KD - NT**	SULT2A1	Sulfotransferase 2A1	0.002642	-0.60	0.66
**KD - NT**	DECR2	Peroxisomal 2,4-dienoyl-CoA reductase	0.002888	0.56	1.48
**KD - NT**	PON1	Serum paraoxonase/arylesterase 1	0.003019	-0.77	0.59
**KD - NT**	PNPLA2	Patatin-like phospholipase domain-containing protein 2	0.003203	-0.69	0.62
**KD - NT**	PLA2G6	Patatin-like phospholipase domain-containing protein 9	0.003506	-0.85	0.55
**KD - NT**	ACOT7	Cytosolic acyl coenzyme A thioester hydrolase	0.003515	0.53	1.44
**KD - NT**	EGR1	Early growth response protein 1	0.003783	0.84	1.78
**KD - NT**	PDK1	3-phosphoinositide-dependent protein kinase 1	0.004379	-0.63	0.64
**KD - NT**	PDK1	Pyruvate dehydrogenase kinase isoform 1	0.004379	-0.63	0.64
**KD - NT**	SLC45A3	Solute carrier family 45 member 3	0.004518	0.88	1.85

Comparison	Symbol	Protein names	P. Val	LogFC	Fold Change
**KD - NT**	PDGFB	Platelet-derived growth factor subunit B	0.005481	1.29	2.45
**KD - NT**	ID2	DNA-binding protein inhibitor ID-2	0.005720	0.77	1.71
**KD - NT**	MED4	Mediator complex subunit 4	0.006551	-0.58	0.67
**KD - NT**	INPP5J	Inositol polyphosphate 5-phosphatase J	0.008313	1.12	2.17
**KD - NT**	AKR1D1	Aldo-keto reductase family 1 member D1	0.008674	1.33	2.52
**KD - NT**	PDGFA	Platelet-derived growth factor subunit A	0.008825	-0.55	0.68
**KD - NT**	APOC3	Apolipoprotein C-III	0.009457	-0.87	0.55
**KD - NT**	GNB3	Guanine nucleotide-binding protein G	0.010164	-0.85	0.55
**KD - NT**	ALB	Albumin	0.010572	0.55	1.46
**KD - NT**	ALB	Fas-binding factor 1	0.010572	0.55	1.46
**KD - NT**	MED7	Mediator of RNA polymerase II transcription subunit 7	0.011572	-0.54	0.69
**KD - NT**	HSD17B8	Estradiol 17-beta-dehydrogenase 8	0.013272	-0.71	0.61
**KD - NT**	LDLR	Low-density lipoprotein receptor	0.015460	0.55	1.47
**KD - NT**	ABHD4	Alpha/beta hydrolase domain-containing protein 4	0.017082	0.56	1.47
**KD - NT**	FDX1	Ferredoxin-1	0.020050	0.56	1.48
**KD - NT**	CIDEC	Cell death activator CIDE-3	0.028195	0.87	1.83
**KD - NT**	CYP2C9	Cytochrome P450 2C9	0.029385	-0.99	0.50
**KD - NT**	ACER3	Alkaline ceramidase 3	0.031183	0.52	1.43
**KD - NT**	AKR1C2	Aldo-keto reductase family 1 member C2	0.037809	1.00	2.00
**KD - NT**	TM6SF2	Transmembrane 6 superfamily member 2	0.428827	-0.29	0.82

Supplementary Table S2: Genes that were differentially expressed **(**adjusted *p* value of less than 0.05 and an absolute log fold change of greater than 1 or 1.4-fold change) in cells with either wild-type *TM6SF2* overexpression or knockdown compared to their controls (vector or non-targeted controls respectively) are shown. WT (wild-type *TM6SF2* overexpression), Vector (empty vector control), NT (Non-targeted shRNA control), and KD (*TM6SF2* knockdown). Only the set of 1008 genes for the 749 proteins identified by Reactome as being part of the overall human Metabolism of lipids pathway (R-HSA-556833) andthe 864 human proteins identified in GO as being involved in the regulation of lipid metabolic process (GO:0019216) were analyzed.

Supplementary Method S1: CellProfiler Pipeline

http://www.cellprofiler.org
Version:5
DateRevision:413
GitHash:
ModuleCount:22
HasImagePlaneDetails:False

Images:[module_num:1|svn_version:'Unknown'|variable_revision_number:2|show_window:False|notes:['To begin creating your project, use the Images module to compile a list of files and/or folders that you want to analyze. You can also specify a set of rules to include only the desired files in your selected folders.']|batch_state:array([], dtype=uint8)|enabled:True|wants_pause:False]
:
Filter images?:Custom
Select the rule criteria:and (file does endwith ".C01")

Metadata:[module_num:2|svn_version:'Unknown'|variable_revision_number:6|show_window:False|notes:['The Metadata module optionally allows you to extract information describing your images (i.e, metadata) which will be stored along with your measurements. This information can be contained in the file name and/or location, or in an external file.']|batch_state:array([], dtype=uint8)|enabled:True|wants_pause:False]
Extract metadata?:Yes
Metadata data type:Text
Metadata types:{}
Extraction method count:1
Metadata extraction method:Extract from file/folder names
Metadata source:File name
Regular expression to extract from file name:^(?P<WELL>.*)_(?P<CELL>.*)_(?P<COND>.*)_(?P<srs>.*)_(?P<dye>.*).C01
Regular expression to extract from folder name:(?P<Date>[0-9]{4}_[0-9]{2}_[0-9]{2})$
Extract metadata from:All images
Select the filtering criteria:and (file does contain "")
Metadata file location:Elsewhere...|
Match file and image metadata:[]
Use case insensitive matching?:No
Metadata file name:
Does cached metadata exist?:No

NamesAndTypes:[module_num:3|svn_version:'Unknown'|variable_revision_number:8|show_window:False|notes:[' The NamesAndTypes module allows you to assign a meaningful name to each image by which other modules will refer to it.']|batch_state:array([], dtype=uint8)|enabled:True|wants_pause:False]
Assign a name to:Images matching rules
Select the image type:Grayscale image
Name to assign these images:DNA
Match metadata:[{'Raw_Lipid': 'CELL', 'Raw_DNA': 'CELL'}, {'Raw_Lipid': 'COND', 'Raw_DNA': 'COND'}, {'Raw_Lipid': 'WELL', 'Raw_DNA': 'WELL'}, {'Raw_Lipid': 'srs', 'Raw_DNA': 'srs'}]
Image set matching method:Metadata
Set intensity range from:Image metadata
Assignments count:2
Single images count:0
Maximum intensity:255.0
Process as 3D?:No
Relative pixel spacing in X:1.0
Relative pixel spacing in Y:1.0
Relative pixel spacing in Z:1.0
Select the rule criteria:and (file does endwith "DNA.C01")
Name to assign these images:Raw_DNA

Name to assign these objects:Cell
Select the image type:Grayscale image
Set intensity range from:Image metadata
Maximum intensity:255.0
Select the rule criteria:and (file does endwith "Lipid.C01")
Name to assign these images:Raw_Lipid
Name to assign these objects:Nucleus
Select the image type:Grayscale image
Set intensity range from:Image metadata
Maximum intensity:255.0

Groups:[module_num:4|svn_version:'Unknown'|variable_revision_number:2|show_window:False|notes:['The Groups module optionally allows you to split your list of images into image subsets (groups) which will be processed independently of each other. Examples of groupings include screening batches, microtiter plates, time-lapse movies, etc.']|batch_state:array([], dtype=uint8)|enabled:True|wants_pause:False]
Do you want to group your images?:No
grouping metadata count:1
Metadata category:None

RescaleIntensity:[module_num:5|svn_version:'Unknown'|variable_revision_number:3|show_window:False|notes:[]|batch_state:array([], dtype=uint8)|enabled:True|wants_pause:False]
Select the input image:Raw_DNA
Name the output image:DNA
Rescaling method:Stretch each image to use the full intensity range
Method to calculate the minimum intensity:Custom
Method to calculate the maximum intensity:Custom
Lower intensity limit for the input image:0.0
Upper intensity limit for the input image:1.0
Intensity range for the input image:0.0,1.0
Intensity range for the output image:0.0,1.0
Select image to match in maximum intensity:None
Divisor value:1.0
Divisor measurement:None

RescaleIntensity:[module_num:6|svn_version:'Unknown'|variable_revision_number:3|show_window:False|notes:[]|batch_state:array([], dtype=uint8)|enabled:True|wants_pause:False]
Select the input image:Raw_Lipid
Name the output image:Lipid
Rescaling method:Match the image's maximum to another image's maximum
Method to calculate the minimum intensity:Custom
Method to calculate the maximum intensity:Custom
Lower intensity limit for the input image:0.0
Upper intensity limit for the input image:1.0
Intensity range for the input image:0.0,1.0
Intensity range for the output image:0.0,1.0
Select image to match in maximum intensity:DNA
Divisor value:1.0
Divisor measurement:None

EnhanceOrSuppressFeatures:[module_num:7|svn_version:'Unknown'|variable_revision_number:7|show_window:False|notes:[]|batch_state:array([], dtype=uint8)|enabled:True|wants_pause:False]
Select the input image:Lipid
Name the output image:Enhanced_Lipid
Select the operation:Enhance
Feature size:30
Feature type:Speckles
Range of hole sizes:1,10

Smoothing scale:2.0
Shear angle:0.0
Decay:0.95
Enhancement method:Tubeness
Speed and accuracy:Fast
Rescale result image:Yes

IdentifyPrimaryObjects:[module_num:8|svn_version:'Unknown'|variable_revision_number:14|show_window:False|notes:[]|batch_state:array([], dtype=uint8)|enabled:True|wants_pause:False]
Select the input image:DNA
Name the primary objects to be identified:Nuclei
Typical diameter of objects, in pixel units (Min,Max):50,250
Discard objects outside the diameter range?:Yes
Discard objects touching the border of the image?:Yes
Method to distinguish clumped objects:Intensity
Method to draw dividing lines between clumped objects:Intensity
Size of smoothing filter:10
Suppress local maxima that are closer than this minimum allowed distance:7.0
Speed up by using lower-resolution image to find local maxima?:Yes
Fill holes in identified objects?:After both thresholding and declumping
Automatically calculate size of smoothing filter for declumping?:Yes
Automatically calculate minimum allowed distance between local maxima?:Yes
Handling of objects if excessive number of objects identified:Continue
Maximum number of objects:500
Display accepted local maxima?:No
Select maxima color:Blue
Use advanced settings?:Yes
Threshold setting version:12
Threshold strategy:Global
Thresholding method:Minimum Cross-Entropy
Threshold smoothing scale:2
Threshold correction factor:2
Lower and upper bounds on threshold:0.17,1
Manual threshold:0.0
Select the measurement to threshold with:None
Two-class or three-class thresholding?:Two classes
Log transform before thresholding?:No
Assign pixels in the middle intensity class to the foreground or the background?:Foreground
Size of adaptive window:50
Lower outlier fraction:0.05
Upper outlier fraction:0.05
Averaging method:Mean
Variance method:Standard deviation
# of deviations:2.0
Thresholding method:Otsu

IdentifyPrimaryObjects:[module_num:9|svn_version:'Unknown'|variable_revision_number:14|show_window:False|notes:[]|batch_state:array([], dtype=uint8)|enabled:True|wants_pause:False]
Select the input image:Enhanced_Lipid
Name the primary objects to be identified:Lipid_Droplets
Typical diameter of objects, in pixel units (Min,Max):1,30
Discard objects outside the diameter range?:Yes
Discard objects touching the border of the image?:Yes
Method to distinguish clumped objects:Intensity
Method to draw dividing lines between clumped objects:Propagate
Size of smoothing filter:10
Suppress local maxima that are closer than this minimum allowed distance:7.0

Speed up by using lower-resolution image to find local maxima?:Yes
Fill holes in identified objects?:After both thresholding and declumping
Automatically calculate size of smoothing filter for declumping?:Yes
Automatically calculate minimum allowed distance between local maxima?:Yes
Handling of objects if excessive number of objects identified:Continue
Maximum number of objects:500
Display accepted local maxima?:No
Select maxima color:Blue
Use advanced settings?:Yes
Threshold setting version:12
Threshold strategy:Global
Thresholding method:Minimum Cross-Entropy
Threshold smoothing scale:1
Threshold correction factor:2
Lower and upper bounds on threshold:0.05,0.5
Manual threshold:0.0
Select the measurement to threshold with:None
Two-class or three-class thresholding?:Two classes
Log transform before thresholding?:No
Assign pixels in the middle intensity class to the foreground or the background?:Foreground
Size of adaptive window:50
Lower outlier fraction:0.05
Upper outlier fraction:0.05
Averaging method:Mean
Variance method:Standard deviation
# of deviations:2.0
Thresholding method:Otsu

IdentifySecondaryObjects:[module_num:10|svn_version:'Unknown'|variable_revision_number:10|show_window:False|notes:[]|batch_state:array([], dtype=uint8)|enabled:True|wants_pause:False]
Select the input objects:Nuclei
Name the objects to be identified:Lipid_Cells
Select the method to identify the secondary objects:Propagation
Select the input image:Lipid
Number of pixels by which to expand the primary objects:10
Regularization factor:0.05
Discard secondary objects touching the border of the image?:Yes
Discard the associated primary objects?:Yes
Name the new primary objects:LipidCellNoNuclei
Fill holes in identified objects?:No
Threshold setting version:12
Threshold strategy:Global
Thresholding method:Minimum Cross-Entropy
Threshold smoothing scale:0.5
Threshold correction factor:.9
Lower and upper bounds on threshold:0.06,1.0
Manual threshold:0.0
Select the measurement to threshold with:None
Two-class or three-class thresholding?:Two classes
Log transform before thresholding?:No
Assign pixels in the middle intensity class to the foreground or the background?:Foreground
Size of adaptive window:50
Lower outlier fraction:0.05
Upper outlier fraction:0.05
Averaging method:Mean
Variance method:Standard deviation
# of deviations:2.0

Thresholding method:Otsu

IdentifyTertiaryObjects:[module_num:11|svn_version:'Unknown'|variable_revision_number:3|show_window:False|notes:[]|batch_state:array([], dtype=uint8)|enabled:True|wants_pause:False]
Select the larger identified objects:Lipid_Cells
Select the smaller identified objects:Nuclei
Name the tertiary objects to be identified:perimeter
Shrink smaller object prior to subtraction?:Yes

RelateObjects:[module_num:12|svn_version:'Unknown'|variable_revision_number:5|show_window:False|notes:[]|batch_state:array([], dtype=uint8)|enabled:True|wants_pause:False]
Parent objects:Lipid_Cells
Child objects:Lipid_Droplets
Calculate child-parent distances?:None
Calculate per-parent means for all child measurements?:Yes
Calculate distances to other parents?:No
Do you want to save the children with parents as a new object set?:Yes
Name the output object:RelateObjects_lipidCellLipidDroplets
Parent name:None
Parent name:None
Parent name:None
Parent name:None
Parent name:None
Parent name:None
Parent name:None
Parent name:None
Parent name:None
Parent name:None
Parent name:None
Parent name:None
Parent name:None
Parent name:None
Parent name:None
Parent name:None
Parent name:None
Parent name:None
Parent name:None
Parent name:None
Parent name:None
Parent name:None
Parent name:None
Parent name:None
Parent name:None
Parent name:None
Parent name:None
Parent name:None
Parent name:None

MeasureObjectIntensity:[module_num:13|svn_version:'Unknown'|variable_revision_number:4|show_window:False|notes:[]|batch_state:array([], dtype=uint8)|enabled:True|wants_pause:False]
Select images to measure:Raw_Lipid
Select objects to measure:Lipid_Cells, Lipid_Droplets

MeasureObjectIntensity:[module_num:14|svn_version:'Unknown'|variable_revision_number:4|show_window:False|notes:[]|batch_state:array([], dtype=uint8)|enabled:True|wants_pause:False]
Select images to measure:Lipid
Select objects to measure:Lipid_Cells, Lipid_Droplets

MeasureImageIntensity:[module_num:15|svn_version:'Unknown'|variable_revision_number:4|show_window:False|notes:[]|batch_state:array([], dtype=uint8)|enabled:True|wants_pause:False]
    Select images to measure:Lipid
    Measure the intensity only from areas enclosed by objects?:Yes
    Select input object sets:Lipid_Cells
    Calculate custom percentiles:No
    Specify percentiles to measure:10,90

CalculateMath:[module_num:16|svn_version:'Unknown'|variable_revision_number:3|show_window:False|notes:[]|batch_state:array([], dtype=uint8)|enabled:True|wants_pause:False]
    Name the output measurement:Total_Frame_Lipid_Intensity_per_Cell
    Operation:Divide
    Select the numerator measurement type:Image
    Select the numerator objects:None
    Select the numerator measurement:Intensity_MeanIntensity_Lipid_Lipid_Cells
    Multiply the above operand by:1.0
    Raise the power of above operand by:1.0
    Select the denominator measurement type:Object
    Select the denominator objects:Nuclei
    Select the denominator measurement:Number_Object_Number
    Multiply the above operand by:1.0
    Raise the power of above operand by:1.0
    Take log10 of result?:No
    Multiply the result by:1.0
    Raise the power of result by:1.0
    Add to the result:0.0
    How should the output value be rounded?:Not rounded
    Enter how many decimal places the value should be rounded to:0
    Constrain the result to a lower bound?:No
    Enter the lower bound:0.0
    Constrain the result to an upper bound?:No
    Enter the upper bound:1.0

GrayToColor:[module_num:17|svn_version:'Unknown'|variable_revision_number:4|show_window:False|notes:[]|batch_state:array([], dtype=uint8)|enabled:True|wants_pause:False]
    Select a color scheme:RGB
    Rescale intensity:No
    Select the image to be colored red:Leave this black
    Select the image to be colored green:Lipid
    Select the image to be colored blue:DNA
    Name the output image:MergedColorImage
    Relative weight for the red image:1.0
    Relative weight for the green image:1.0
    Relative weight for the blue image:1.0
    Select the image to be colored cyan:Leave this black
    Select the image to be colored magenta:Leave this black
    Select the image to be colored yellow:Leave this black
    Select the image that determines brightness:Leave this black
    Relative weight for the cyan image:1.0
    Relative weight for the magenta image:1.0
    Relative weight for the yellow image:1.0
    Relative weight for the brightness image:1.0
    Hidden:1
    Image name:None
    Color:#ff0000
    Weight:1.0

OverlayOutlines:[module_num:18|svn_version:'Unknown'|variable_revision_number:4|show_window:False|notes:[]|batch_state:array([], dtype=uint8)|enabled:True|wants_pause:False]
Display outlines on a blank image?:Yes
Select image on which to display outlines:None
Name the output image:ObjectOutlines
Outline display mode:Color
Select method to determine brightness of outlines:Max of image
How to outline:Thick
Select outline color:#0080FF
Select objects to display:Nuclei
Select outline color:yellow
Select objects to display:Lipid_Cells
Select outline color:green
Select objects to display:Lipid_Droplets

Tile:[module_num:19|svn_version:'Unknown'|variable_revision_number:1|show_window:True|notes:[]|batch_state:array([], dtype=uint8)|enabled:True|wants_pause:False]
Select an input image:DNA
Name the output image:TiledResults
Tile assembly method:Within cycles
Final number of rows:1
Final number of columns:3
Image corner to begin tiling:top left
Direction to begin tiling:row
Use meander mode?:No
Automatically calculate number of rows?:No
Automatically calculate number of columns?:No
Select an additional image to tile:Enhanced_Lipid
Select an additional image to tile:ObjectOutlines

RescaleIntensity:[module_num:20|svn_version:'Unknown'|variable_revision_number:3|show_window:False|notes:[]|batch_state:array([], dtype=uint8)|enabled:True|wants_pause:False]
Select the input image:TiledResults
Name the output image:RescaledTiledResults
Rescaling method:Match the image's maximum to another image's maximum
Method to calculate the minimum intensity:Custom
Method to calculate the maximum intensity:Custom
Lower intensity limit for the input image:0.0
Upper intensity limit for the input image:1.0
Intensity range for the input image:0.0,1.0
Intensity range for the output image:0.0,1.0
Select image to match in maximum intensity:DNA
Divisor value:1.0
Divisor measurement:None

SaveImages:[module_num:21|svn_version:'Unknown'|variable_revision_number:15|show_window:False|notes:[]|batch_state:array([], dtype=uint8)|enabled:False|wants_pause:False]
Select the type of image to save:Image
Select the image to save:RescaledTiledResults
Select method for constructing file names:Single name
Select image name for file prefix:None
Enter single file name:\g<CELL>_\g<COND>_\g<WELL>_\g<srs>
Number of digits:4
Append a suffix to the image file name?:No
Text to append to the image name:
Saved file format:tiff

Output file location:Elsewhere...|E:\\Speliotes\\Lipid_Staining_Pipeline\\TM6SF2\\2020_02_06_TM6SF2_KD_Nof1\\CellProfilerResultsRenamedCorrectlyRun1
Image bit depth:8-bit integer
Overwrite existing files without warning?:Yes
When to save:Every cycle
Record the file and path information to the saved image?:Yes
Create subfolders in the output folder?:No
Base image folder:Elsewhere...|
How to save the series:T (Time)

ExportToSpreadsheet:[module_num:22|svn_version:'Unknown'|variable_revision_number:13|show_window:False|notes:[]|batch_state:array([], dtype=uint8)|enabled:True|wants_pause:False]
Select the column delimiter:Comma (",")
Add image metadata columns to your object data file?:Yes
Add image file and folder names to your object data file?:No
Select the measurements to export:No
Calculate the per-image mean values for object measurements?:Yes
Calculate the per-image median values for object measurements?:No
Calculate the per-image standard deviation values for object measurements?:No
Output file location:Elsewhere...|E:\\Speliotes\\Lipid_Staining_Pipeline\\TM6SF2\\2020_02_06_TM6SF2_KD_Nof1\\CellProfilerResultsRenamedCorrectlyRun1\\Excel
Create a GenePattern GCT file?:No
Select source of sample row name:Metadata
Select the image to use as the identifier:None
Select the metadata to use as the identifier:None
Export all measurement types?:Yes
Press button to select measurements:
Representation of Nan/Inf:NaN
Add a prefix to file names?:Yes
Filename prefix:V3_
Overwrite existing files without warning?:Yes
Data to export:Do not use
Combine these object measurements with those of the previous object?:No
File name:DATA.csv
Use the object name for the file name?:Yes
